# The power of natural phenolic compounds: caffeic acid is able to enhance the retinoid-induced differentiation of tumor cells

**DOI:** 10.1186/2049-3002-2-S1-P81

**Published:** 2014-05-28

**Authors:** Renata Veselska, Petr Chlapek, Miroslava Krzyzankova, Silvia Chovanova, Jakub Neradil, Karel Zitterbart, Jaroslav Sterba

**Affiliations:** 1 Laboratory of Tumor Biology, Department of Experimental Biology, School of Science, Masaryk University, Kotlarska 2, 611 37 Brno, Czech Republic; 2 Department of Pediatric Oncology, University Hospital Brno and School of Medicine, Masaryk University, Cernopolni 9, 613 00 Brno, Czech Republic; 3 Masaryk Memorial Cancer Institute, Zluty kopec 7, 656 53 Brno, Czech Republic

## Background

The induced differentiation of tumor cells represents a very important strategy in modern antineoplastic therapy. Retinoids are the most frequently used group of cell differentiation inducers; however, their toxicity and intrinsic or acquired resistance to retinoids substantially limit their use in clinical protocols. Therefore, special attention has been paid to the combined treatment of cancer cells with retinoids and other compounds that may modulate (enhance or prolong) their antineoplastic effects. For example, combinations of retinoids and bile acids or inhibitors of the lipoxygenases and cyclooxygenases were reported as effective. Our research was focused on the caffeic acid (CA), a widely distributed plant phenolic compound, which is an inhibitor of 5-lipoxygenase.

## Materials and methods

Eight cell lines derived from different types of pediatric solid tumors (neuroblastomas, medulloblastomas, osteosarcomas, and rhabdomyosarcomas) were chosen for this study. All-*trans* retinoic acid (ATRA) in concentration ranging from 0.05 microM to 10 microM was used as an inducer of cell differentiation. ATRA was applied alone or in combination with caffeic acid (CA) at concentrations of 13 and 52 microM; these concentrations of CA corresponded to the plasma levels obtained by its dietary uptake. Changes in cell morphology, proliferation activity, expression of differentiation markers and other cancer-related genes were evaluated in treated cell populations.

## Results

Although the cell lines showed various sensitivity to the ATRA treatment, the concentrations of CA used in this study were capable to enhance these antineoplastic effects of ATRA, especially in terms of cell differentiation.

## Conclusions

Our results clearly showed that CA, which belongs to the natural phenolic compounds and is present in honey, apple juice, grapes and some other fruits and vegetables, may potentiate the cell differentiation of tumor cells induced by retinoids. These findings were confirmed using gene expression analysis that showed an increasing expression of genes involved in the process of the induced differentiation.

